# Three mammalian tropomyosin isoforms have different regulatory effects on nonmuscle myosin-2B and filamentous β-actin *in vitro*

**DOI:** 10.1074/jbc.M117.806521

**Published:** 2017-11-30

**Authors:** Salma Pathan-Chhatbar, Manuel H. Taft, Theresia Reindl, Nikolas Hundt, Sharissa L. Latham, Dietmar J. Manstein

**Affiliations:** From the ‡ Institute for Biophysical Chemistry and; the § Division for Structural Biochemistry, Hannover Medical School, 30625 Hannover, Germany

**Keywords:** actin, cytoskeleton, molecular motor, myosin, tropomyosin, isoenzyme

## Abstract

The metazoan actin cytoskeleton supports a wide range of contractile and transport processes. Recent studies have shown how the dynamic association with specific tropomyosin isoforms generates actin filament populations with distinct functional properties. However, critical details of the associated molecular interactions remain unclear. Here, we report the properties of actomyosin–tropomyosin complexes containing filamentous β-actin, nonmuscle myosin-2B (NM-2B) constructs, and either tropomyosin isoform Tpm1.8cy (b.–.b.d), Tpm1.12br (b.–.b.c), or Tpm3.1cy (b.–.a.d). Our results show the extent to which the association of filamentous β-actin with these different tropomyosin cofilaments affects the actin-mediated activation of NM-2B and the release of the ATP hydrolysis products ADP and phosphate from the active site. Phosphate release gates a transition from weak to strong F-actin–binding states. The release of ADP has the opposite effect. These changes in dominant rate-limiting steps have a direct effect on the duty ratio, the fraction of time that NM-2B spends in strongly F-actin–bound states during ATP turnover. The duty ratio is increased ∼3-fold in the presence of Tpm1.12 and 5-fold for both Tpm1.8 and Tpm3.1. The presence of Tpm1.12 extends the time required per ATP hydrolysis cycle 3.7-fold, whereas it is shortened by 27 and 63% in the presence of Tpm1.8 and Tpm3.1, respectively. The resulting Tpm isoform–specific changes in the frequency, duration, and efficiency of actomyosin interactions establish a molecular basis for the ability of these complexes to support cellular processes with widely divergent demands in regard to force production, capacity to move processively, and speed of movement.

## Introduction

Tropomyosins (Tpm)^4^ form a large family of double-stranded α-helical coiled-coil actin-binding proteins. They are an integral part of most actin filaments in animals. Over 40 Tpm isoforms are produced by alternate promoter selection and splicing of four different genes: *TPM1*, *TPM2*, *TPM3*, and *TPM4*. Direct differences between the coding regions of *TPM1–4* and alternative usage of variant exons 1, 2, 6, and 9 contribute to Tpm isoform diversity (1). The resulting gene products can be grouped in high-molecular-weight and low-molecular-weight isoforms. High-molecular-weight isoforms are encoded by nine exons and bind seven consecutive actin subunits along the actin long-pitch helix. Low-molecular-weight isoforms lack sequences encoded by exon 2 and interact only with six actin subunits. Although most Tpm isoforms show a wider tissue distribution (2), they are frequently referred to according to their dominant localization as striated muscle, smooth muscle, brain, or cytoskeletal isoforms.

In the sarcomere of striated muscles, isoforms Tpm1.1, Tpm1.2, Tpm2.2, and Tpm3.12 form a complex with troponin that regulates cardiac and skeletal muscle myosin-2 motor activity in a Ca^2+^-dependent manner (3). In smooth muscle cells, Tpm1.3, Tpm1.4, and Tpm2.1 play a more modulatory role in regulating contraction (4). Cytoskeletal tropomyosins (*e.g.* Tpm1.7, Tpm1.8, Tpm1.12, Tpm3.1, and Tpm4.2) represent the largest group of Tpm isoforms. They have been shown to critically affect a wide range of actomyosin-dependent processes including endo- and exocytosis events, the formation and dynamics of cell-surface extensions, rigidity sensing, cell migration, cytokinesis, nuclear division, anchorage-dependent growth, and embryogenesis (5–7). Additionally, changes in the abundance of specific cytoskeletal Tpm isoforms are commonly observed in cells that are undergoing transformation (8). In skeletal and cardiac muscle, the troponin-induced azimuthal translocation of Tpm across the F-actin surface, which controls access of the myosin-binding sites on F-actin, is favored by the low energy cost of the translocation between the A and M states (9–11) and depends critically on tight control of the sarcomeric Ca^2+^ concentration as well as the short duration of strong actin-bound myosin states relative to the overall duration of the reaction cycle (12). Based on a fairly complete analysis of the chemomechanical properties for most of the common myosin isoforms (13–16) and reports showing that cooperative units extend beyond single Tpm dimer boundaries (17, 18), it appears unlikely that cytoskeletal and smooth muscle Tpm isoforms play a similar regulatory role as gatekeeper of the myosin-binding sites on actin. Head-to-tail interactions and the formation of a continuous cable contribute critically to the extension of cooperative units and enhance the interaction of all Tpm isoforms with the filamentous actin (F-actin) substrate (19). Continuous Tpm cables bind to F-actin with ∼1000-fold higher affinity than individual Tpm molecules (20). Structural studies show how the C-terminal coiled-coil region of the Tpm dimer opens over the last 11–15 amino acids, whereas the N-terminal coiled-coil inserts into the resulting cleft (21–24). In the case of muscle Tpm isoforms, *N*-acetylation of the N-terminal methionine contributes critically to the formation and stabilization of a stable overlap complex. In contrast, the ability of Tpm isoforms to interact with F-actin appears to be less critically affected by N-terminal acetylation. Thus, the effect of a lack of *N*-acetylation of the skeletal muscle isoform Tpm1.1 on actin binding is compensated by the replacement of exons 1a and 2b with the N-terminal exon 1b that is present in cytoskeletal isoforms Tpm1.8, Tpm1.12, or Tpm3.1 (25). Moreover, subtle changes near the N terminus appear to have a critical effect on head-to-tail interactions. The ability of muscle Tpm isoforms to polymerize and efficiently bind to F-actin has been reported to be enhanced by the addition of a single glycine residue, an Ala-Ser dipeptide, or a Gly-Ala-Ser tripeptide to their N termini (24, 26, 27). Exon 9-encoded sequences are another major determinant in regard to head-to-tail interactions. Replacement of the striated muscle specific exon 9a encoded C terminus with exon 9d, which is found in smooth muscle and cytoskeletal Tpm isoforms, allows nonacetylated hybrid Tpm to efficiently bind to F-actin (28).

Nonmuscle myosin-2 (NM-2) isoforms are among the most prominent members of the myosin family that associate with Tpm-enveloped cytoskeletal F-actin (29, 30). In mammals there are three genes (*MYH9*, *MYH10*, and *MYH14*) encoding NM-2A, NM-2B, and NM-2C heavy chains, respectively. So-called NM-2 monomers consist of two heavy chains, each associated with an essential and a regulatory myosin light chain. Phosphorylation of the regulatory light chain is required for the monomers to unfold from an autoinhibitory conformation, acquire motor activity, and gain the ability to form bipolar thick filaments that typically contain 20–30 monomers (31, 32). At least in the case of NM-2A and NM-2B, both activated monomers and bipolar thick filaments appear to contribute to distinct cellular functions (33). Advances in the structural characterization of large protein complexes by electron cryomicroscopy have contributed new and important insights into actin–Tpm–myosin complexes (34). Structures resolved to subnanometer scale show that Tpm forms a 210 Å^2^ interaction surface with F-actin and a 300 Å^2^ interaction surface with the myosin motor domain (9, 35). Based on these results, it is possible to begin to relate known differences in their interactions (4) to the structural features of individual myosin and Tpm isoforms. Cytoskeletal Tpm isoforms specify functionally distinct F-actin populations acting as selectivity filters that modulate allosteric coupling and mediate the isoform-specific recruitment of myosins in the context of stress fibers, transverse arcs, and other actin-based superstructures (37, 38). The modulation of myosin motor activity by cytoskeletal Tpm isoforms is not restricted to changes in actin affinity but extends to the kinetics of individual steps in the ATP hydrolysis cycle (39–42). The nature of the M-Tpm contact areas suggests that myosin can greatly enhance Tpm binding to F-actin (10), as was previously shown for a series of *Escherichia coli* expressed variants of rat α-tropomyosin (25).

In this study, we characterized the kinetic and motor properties of complexes composed of filamentous β-actin, NM-2B, and either Tpm1.8cy (b.–.b.d), Tpm1.12br (b.–.b.c), and Tpm3.1cy (b.–.a.d). Tpm3.1 is encoded by TPM3 and contains three regions that differ from the corresponding regions in Tpm1.8 and Tpm1.12. In addition to the changes introduced by the use of TPM3 exon 6a, differences occur in the regions encoded by exons 1 and 9. The regions encoded by exons 1b and 6a contain 5 and 4 charge changes compared with Tpm1.8 and Tpm1.12, respectively. Exon 9d contains 3 charge changes compared with Tpm1.8 and 11 charge changes compared with Tpm1.12. The program Paircoil2 (43) predicts that only the C-terminal ends of Tpm1.8 and Tpm1.12 are affected by differences in their amino acid sequence. In the case of Tpm3.1, Paircoil2 indicates a slightly reduced likelihood of coiled-coil formation in the region from residues Val^134^ to Cys^170^. The organization of the C-terminal end of Tpm3.1 is predicted to more closely resemble Tpm1.8.

## Results

### NM-2B and Tpm isoforms in neuroblastoma cells

**Figure 1.**
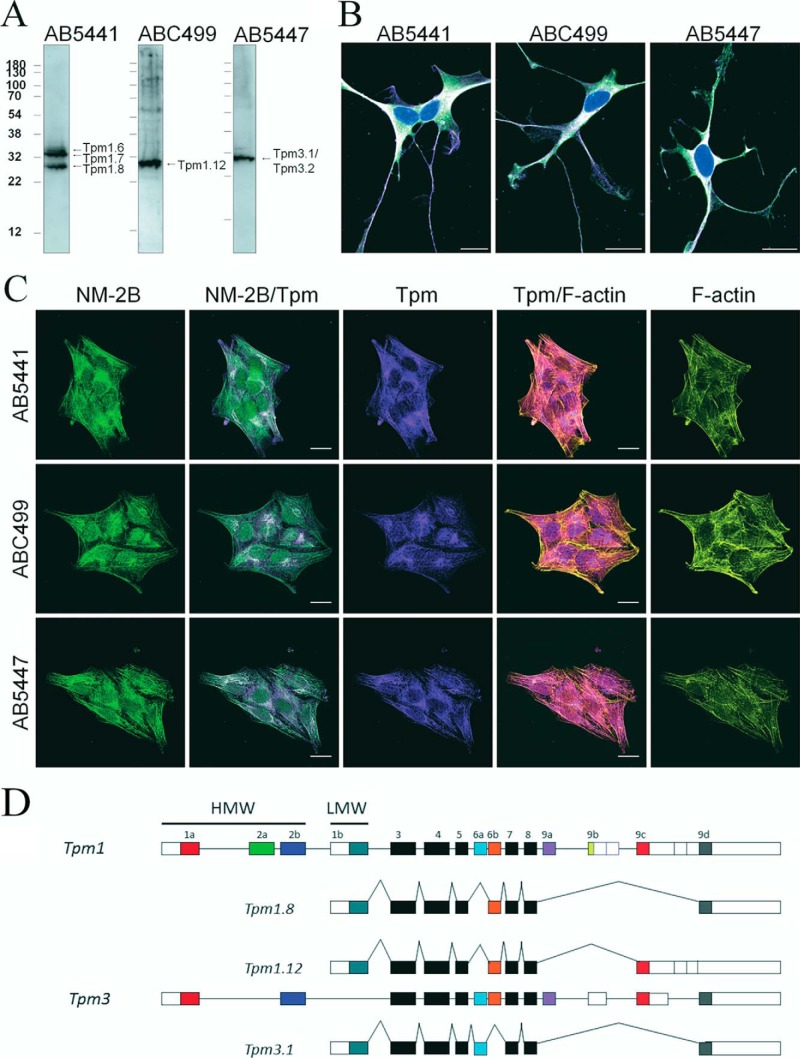
**Tpm1.8, Tpm1.12, and Tpm3.1 are prominently produced and widely distributed in differentiated neuroblastoma cells.** *A*, human neuroblastoma cell line SK-N-BE(2) was exposed for 6 days to 10 μm all-*trans*-retinoic acid to obtain a distinct neuronal phenotype. Immunoblot of differentiated SK-N-BE(2) cells. *B*, stress fibers and thick actin bundles are not prominent in the 6-day differentiated SK-N-BE(2) cells. *C*, SK-N-BE(2) cells differentiated for 2 days display a neuro-epithelial phenotype where bundled actin structures are prominent. Regions where NM-2B and the respective Tpm antibodies colocalize are observed under both conditions, supporting that these are physiological combinations. The sheep polyclonal antibodies used are specific for select tropomyosin isoforms: AB5441 (*left panel*) recognizes Tpm1.6 (32,606 g/mol), Tpm1.7 (32,745 g/mol), and Tpm1.8 (28,477 g/mol); ABC499 recognizes Tpm1.12 (28,254 g/mol); AB447 recognizes both Tpm3.1 (28,902 g/mol) and Tpm3.2 (28,739 g/mol). The cells on coverslips were fixed and stained for different combinations of cytoskeletal Tpm (*magenta*) with NM-2B (*green*). The nuclei were stained with DAPI (*blue*). Areas with high concentrations of both NM-2B and one of the three Tpm isoforms appear *white. Scale bars*, 20 μm. *D*, schematic representation of the exon usage for Tpm1.8, Tpm1.12, and Tpm3.1. Alternative splicing generates products with different N termini, the mutually exclusive presence of 6a or 6b, and alternative C termini. Color coding is used to indicate that exons 1a, 2b, 6a, 6b, 9c, and 9d from the *Tpm1* gene are more similar to their *Tpm3* counterparts than to alternative exons from the *Tpm1* gene. *Black* and *open boxes* indicate invariant and regulatory untranslated regions, respectively. *HMW*, high molecular weight; *LMW*, low molecular weight.

NM-2B is the dominant NM-2 isoform in neuronal cells (44). In differentiated SK-N-BE(2) neuroblastoma cells, NM-2B is widely distributed but somewhat enriched within the cell body and in axonal projections. The abundance and localization of specific Tpm isoforms in neuroblastoma cells can be assessed using antibodies that recognize peptides encoded by exons 1b, 9c, and 9d of *TPM1* and exon 9d of the *TPM3* (1). Each of these antibodies recognizes multiple Tpm isoforms as summarized in Table 1. Using this approach, Gunning and co-workers (45) revealed the presence of Tpm1.10, Tpm1.11, Tpm1.12, and Tpm4.2 in neuroblastoma cells. Similar to most tumor cells, neuroblastoma cells do also produce Tpm3.1. Our results show that in addition, Tpm1.6, Tpm1.7, Tpm1.8, and Tpm3.2 are produced in these cells (Fig. 1*A*). Immunofluorescence staining revealed partially overlapping localization patterns for the different Tpm isoforms and strong colocalization with NM-2B (Fig. 1*B*). Stress fibers that are prominent before the induction of differentiation are difficult to detect at the final stage but can be identified in cells with a neuroepithelial phenotype after 2 days of differentiation (Fig. 1*C*). Any observed differences between Tpm1.8 and Tpm1.12 are caused by differences in amino acid sequence occurring in the C-terminal region, which is encoded by exons 9d and 9c, respectively (Fig. 1*D*). Tpm1.8 carries a C-terminal Asn-Asn-Met tripeptide extension. The preceding 23 residues differ in 18 positions, with 15 changes corresponding to non-conservative substitutions. Tpm3.1 is encoded by *Tpm3* and differs in 58 and 62 residues from Tpm1.8 and Tpm1.12, respectively. Clusters of non-conservative substitutions are located in the region between residues 155 and 175 and near to the C terminus. In addition, 18 substitutions occur within the 50 N-terminal residues, including 5 that lead to charge changes.

**Table 1:** **Exon usage, epitope recognition by antibodies, and size of Tpm isoforms** The bold type indicates the Tpm isoforms characterized in this work.

Isoform*^a^*	Alternate name	Gene	Exon usage*^b^*	Epitope/antibody	Molecular mass*^c^*
					*kg/mol*
Tpm1.6cy (a.b.b.d)	Tm2	*TPM1* (α)	1a.2b.3.4.5.6b.7.8.9d	α9d/AB-5441	32.7
Tpm1.7cy (a.b.a.d)	Tm3 or Tm1.4	*TPM1* (α)	1a.2b.3.4.5.6a.7.8.9d	α9d/AB-5441	32.9
**Tpm1.8cy (b.–.b.d)**	**Tm5a**	***TPM1* (α)**	**1b.–.3.4.5.6b.7.8.9d**	**α9d/AB-5441 α1b/ABC499**	**28.6**
Tpm1.9cy (b.–.a.d)	Tm5b	*TPM1* (α)	1b.–.3.4.5.6a.7.8.9d	α9d/AB-5441 α1b/ABC499	28.8
Tpm1.11br (b.–.b.b)	TmBr2	*TPM1* (α)	1b.–.3.4.5.6b.7.8.9b	α1b/ABC499	28.7
**Tpm1.12br (b.–.b.c)**	**TmBr3**	***TPM1* (α)**	**1b.–.3.4.5.6b.7.8.9c**	**α1b/ABC499**	**28.4**
**Tpm3.1cy (b.–.a.d)**	**Tm5NM1**	***TPM3* (γ)**	**1b.–.3.4.5.6a.7.8.9d**	**γ9d/AB5447**	**29.0**
Tpm3.2cy (b.–.b.d)	Tm5NM2	*TPM3* (γ)	1b.–.3.4.5.6b.7.8.9d	γ9d/AB5447	28.9

*^a^* Nomenclature according to Ref. 2.

*^b^* Exon usage according to Ref. 3.

*^c^* Calculated molecular mass of single polypeptide chain.

### Cooperative binding of Tpm and NM-2B to filamentous β-actin

**Figure 2.**
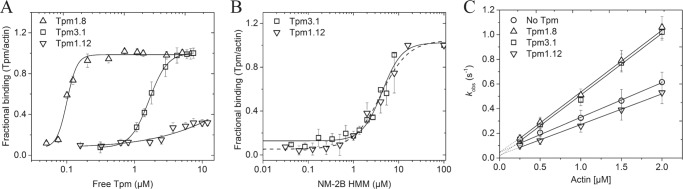
**Binding of cytoskeletal Tpm isoforms and NM-2B to filamentous β-actin and interdependence of Tpm and NM-2B binding.** *A*, binding curves for the interaction of Tpm 1.8, Tpm3.1, and Tpm1.12 with filamentous β-actin. The data were fitted with Hill's equation, where the fractional binding (Tpm/actin) was normalized to distribute between 0 (unbound) and 1 (fully bound). The apparent equilibrium dissociation constants obtained from the fit are listed in Table 2. *B*, NM-2B–HMM-mediated enhancement of Tpm-binding to F-actin. Complex formation of actin-Tpm-NM-2B–HMM was analyzed in sedimentation experiments after the mixing of 6 μm β-actin or 1 μm Tpm1.12 or Tpm3.1 in the presence of 0–16 μm NM-2B. Best-fit curves to the Hill equation are shown as a *dashed line* for Tpm1.12 and a *solid line* for Tpm3.1. Half-fractional saturation with Tpm3.1 and Tpm1.12 is reached in the presence of 4.3 ± 0.5 and 4.1 ± 0.5 μm NM-2B–HMM, respectively. *C*, the observed rate constants for NM-2B binding to bare and Tpm-enveloped β-actin are plotted against the actin concentration. The second-order rate constants for actin binding were obtained from linear fits to the data and are listed in Table 2. The occupancy of β-actin with Tpm1.12 in this experiment (preincubation in the absence of NM-2B–HMM) is in the range of ∼30%; thus the observed reduction of the actin binding rate can be assumed to be more pronounced under cellular conditions. The data are expressed as the means ± S.E. The data shown in *A* and *B* were obtained from four independent measurements. The data shown in *C* are the average of six measurements and were reproduced at least once with a different batch of protein.

We performed cosedimentation assays to determine binding affinities for the interaction between filamentous β-actin and individual Tpm isoforms. Our results show that in the absence of NM-2B, Tpm1.12 binds filamentous β-actin with much lower affinity than Tpm1.8 and Tpm3.1. The corresponding *K*_50%_ values correspond to ≥40 μm (Tpm1.12), 1.6 ± 0.2 μm (Tpm3.1), and 0.10 ± 0.03 μm (Tpm1.8) (Fig. 2*A*). Similar results were described in previous reports performed with α-actin (25, 46). Specifically, Moraczewska *et al.* (25) showed that chicken skeletal muscle myosin subfragment-1 greatly increases the affinity of Tpm1.12 and other cytoskeletal Tpm variants for F-actin to biologically significant levels. In our experiments, the presence of NM-2B–HMM increased the affinity of the Tpm isoforms for filamentous β-actin to the extent that complete decoration of F-actin was observed in mixtures containing 6 μm β-actin and 1 μm Tpm1.12 or Tpm3.1. Half-fractional saturation is reached in the presence of 4.3 ± 0.5 μm NM-2B–HMM for Tpm3.1 and 4.1 ± 0.5 μm NM-2B–HMM in the case of Tpm1.12 (Fig. 2*B*). Full-fractional saturation with Tpm1.8 is already reached with nanomolar concentrations of NM-2B–HMM. The high affinity of Tpm1.8 for F-actin interferes with a more detailed analysis of the cooperative enhancement of actin-binding in the presence of NM-2B–HMM, because the linearity of the sedimentation assay is compromised under these conditions.

The rate of NM-2B binding to filamentous β-actin was assessed by monitoring the transient increase in light scattering upon mixing of the single-headed construct NM-2B0 (47) with either bare or Tpm-enveloped F-actin. In each case, the transients followed a single-exponential function. A secondary plot of the observed rate constants against the concentration of bare or Tpm-enveloped F-actin revealed a linear dependence for each combination of proteins (Fig. 2*C*). This is compatible with a simple one-step binding process, where *k*_obs_ = [A] *k*_+_*_A_* + *k*_−_*_A_* (48). The value of the second-order association rate constant *k*_+_*_A_* is given by the slope of the plot. Decoration with Tpm1.12 led to a 20% reduction in *k*_+_*_A_*, whereas an ∼50% increase in *k*_+_*_A_* was observed with Tpm1.8 and Tpm3.1. The *y* intercept was used to estimate the first-order dissociation rate constant k_−_*_A_*. The affinity of NM-2B0 for filamentous β-actin (*K_A_*) was calculated from the ratio *k*_−_*_A_*/*k*_+_*_A_*_._ The results are summarized in Table 2.

**Table 2:** **Protein–protein interactions and steady-state and transient kinetics** NA, not applicable.

	No Tpm	Tpm1.8	Tpm3.1	Tpm1.12
**Acto-myosin binding (in the absence of nucleotide)*^a^***				
*k*_+_*_A_* (μm^−1^ s^−1^)	0.32 ± 0.03	0.51 ± 0.02	0.49 ± 0.02	0.25 ± 0.01
*k*_−_*_A_* (s^−1^)*^b^*	0.02 ± 0.01	0.03 ± 0.01	0.015 ± 0.01	0.02 ± 0.01
*K_A_* (nm)*^c^*	60 ± 30	60 ± 20	30 ± 20	80 ± 10

**Tpm binding**				
*K*_50%_ (μm) (F-actin)	NA	0.1 ± 0.03	1.6 ± 0.2	≥40
*K*_50%_ (μm) (acto-NM-2B)*^d^*	NA	<0.1	<1	<1

**Steady-state ATPase**				
*k*_cat_ (s^−1^)	0.44 ± 0.07	0.56 ± 0.02	0.72 ± 0.07	0.12 ± 0.01
*K*_app_ (μm)	47.51 ± 13.08	19.92 ± 1.67	30.14 ± 5.36	12.16 ± 3.36
*k*_cat_/*K*_app_ (μm^−1^ s^−1^)*^e^*	0.007 ± 0.001	0.020 ± 0.001	0.018 ± 0.001	0.006 ± 0.001

**ADP release*^f^***				
*k*_+_ (assisting load ) (s^−1^)*^g^*	0.51 ± 0.05	0.18 ± 0.07	0.24 ± 0.08	0.14 ± 0.06
*k*_−_ (resisting load) (s^−1^)*^h^*	0.023 ± 0.001	0.001 ± 0.0005	0.007 ± 0.003	0.003 ± 0.0005

**Phosphate release***^i^*				
*k*_obs_ (s^−1^)	0.078 ± 0.02	0.41 ± 0.09	18.7 ± 2.2	0.13 ± 0.03

**Motor properties**				
Sliding velocity (nm s^−1^)	16.3 ± 5.6	15.1 ± 3.6	10.3 ± 2.9	11.7 ± 4.2
Velocity, TIRF assay (nm s^−1^)	5.3 ± 0.2	7.2 ± 0.4	7.1 ± 0.4	NA
*n*, landing assay	5.0 ± 1.2	0.9 ± 0.1	1.0 ± 0.2	1.5 ± 0.4

*^a^* Measured in the presence of 100 mm KCl.

*^b^* Determined from the *y* intercept.

*^c^* Calculated value (*k*_−_*_A_*/*k*_+_*_A_*).

*^d^* In the presence of 5 μm NM-2B-HMM.

*^e^* The apparent second-order rate constant *k*_cat_*/K*_app_ was obtained from the initial slope of the steady-state ATPase activity versus actin concentration plot.

*^f^* Post mix concentrations: 0.2 μm myosin heads, 5 μm dmADP, 1 μm actin, 1 mm ADP, and 100 mm KCl.

*^g^* Trailing head.

*^h^* Leading head.

*^i^* Measured at 20 μm β-actin.

### Modulation of the actin-activated ATPase activity and duty ratio of NM-2B by Tpm isoforms

**Figure 3.**
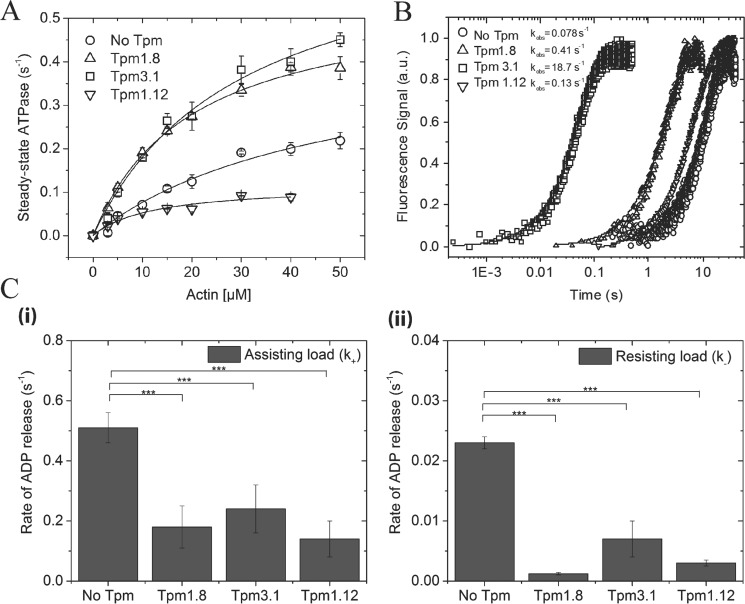
**Kinetic analysis of Tpm-induced changes in ATP turnover of acto-NM-2B–HMM and the release of the hydrolysis products ADP and P_i_.** *A*, actin-activated ATPase activity of NM-2B–HMM measured at β-actin concentrations in the range from 0 to 50 μm. For Tpm-enveloped actin, 3-fold molar access of Tpm was used at each actin concentration. Whereas for Tpm1.12, the occupancy at the lowest actin concentration (3 μm actin, 9 μm Tpm1.12) is only ∼30% and increases to a maximum of 40% at the highest actin concentration used (40 μm actin, 120 μm Tpm1.12), the occupancy for both Tpm1.8 and Tpm3.1 is 100% for all actin concentrations. The observed rates were plotted against the actin concentration and fitted to a hyperbola. The values for the maximum actin-activated ATPase activity (*k*_cat_), the actin concentration required for half-maximal activation (*K*_app_), and the apparent second-order rate constant for F-actin binding (*k*_cat_/*K*_app_) are summarized in Table 2. *B*, rate of P_i_ release from acto-NM-2B–HMM in the presence and absence of Tpm1.8. At the conditions used (20 μm actin, 60 μm Tpm), Tpm exhibits an occupancy of ∼40% for Tpm1.12 and 100% for both Tpm1.8 and Tpm3.1. The single turnover trace was obtained in double-mixing experiments. The ensuing increase in the fluorescence of the phosphate sensor *N*-[2-(1-maleimidyl)ethyl]-7-(diethylamino)coumarin-3-carboxamide–labeled phosphate-binding protein was fitted to a single exponential in all cases. No initial burst was observed before the onset of the steady-state period. *C*, Tpm-induced changes in ADP release from acto-NM-2B–HMM were monitored by following the decrease in dmADP fluorescence upon mixing acto-NM-2B–HMM complexes with a large excess of unlabeled ADP in the presence or absence of Tpm. Post mix concentrations in the stopped-flow apparatus correspond to 0.2 μm myosin heads, 5 μm dmADP, 1 μm actin, 1 mm ADP, and either 0 or 10 μm Tpm construct. The transients were best fit to double exponential functions yielding *k*_obs_ values. The values obtained for the fast phase are shown in *panel (i)* and for the slow phase in *panel (ii)*. The data are expressed as the means ± S.E. Each point represents the average of five to six reactions and three replicate series, and the results were reproduced in three batches of protein.

The actin-activated Mg^2+^ATPase activity of myosin light chain kinase (MLCK)-treated NM-2B–HMM was measured with bare- and Tpm-enveloped filamentous β-actin. Decoration with Tpm1.8 or Tpm3.1 increases the maximum actin-activated ATPase activity (*k*_cat_) of NM-2B–HMM 1.3- and 1.6-fold, respectively, whereas Tpm1.12 reduces *k*_cat_ 3.6-fold (Fig. 3*A*). The actin concentration required for half-maximal activation (*K*_app_) is significantly reduced by all three Tpm isoforms (Table 2). It should be noted that the individual values for *k*_cat_ and *K*_app_ are only estimates and must be treated with some caution. Reliable readings were obtained only up to 50 μm enveloped F-actin, which is in the order of *K*_app_. In contrast, the values shown in Table 2 for the apparent second-order rate constant *k*_cat_/*K*_app_ are well defined by the initial slope of the ATPase activity *versus* [F-actin] plot. They reflect the behavior of the fully activated complex and are a measure of the coupling efficiency between the actin and nucleotide-binding sites of myosin (49). The coupling efficiency increases 2.9-fold in the presence of Tpm1.8 and 2.5-fold in the presence of Tpm3.1 but is 14.3% reduced in the presence of Tpm1.12 (Fig. 3*A* and Table 2).

To determine the influence of Tpm isoforms on individual steps of the NM-2B ATP-turnover cycle, we performed a series of stopped-flow experiments. In a first step, we measured the effect of Tpm decoration on the rate of P_i_ release from acto-NM-2B–HMM. Compared with bare F-actin (*k*_obs_ = 0.078 ± 0.02 s^−1^), we observed ∼5.3- and 240-fold acceleration of the release of inorganic phosphate (P_i_) from the active site of NM-2B in the presence of Tpm1.8 (0.41 ± 0.09 s^−1^) and Tpm3.1 (18.7 ± 2.2 s^−1^), respectively. In contrast, Tpm1.12 only marginally affected the P_i_ release rate of the NM-2B construct (0.13 ± 0.03 s^−1^) (Fig. 3*B* and Table 2).

Next, we evaluated the rate of ADP release from acto-NM-2B–HMM using 2′-deoxy-3′-mant-ADP (dmADP) (50). This approach exploits the decrease of mant-fluorescence that occurs when dmADP is displaced from acto-NM-2B–HMM in the presence of excess ADP. The ensuing transients display biphasic behavior. Sellers and co-workers (51) assigned the fast phase to ADP release from the trailing head, which experiences a forward assisting load, and the slow phase to ADP release from the leading head, which experiences a resisting load. The values obtained for the fast phase are 0.51 ± 0.05 s^−1^ for bare actin, 0.18 ± 0.07 s^−1^ for Tpm1.8-actin, 0.24 ± 0.08 s^−1^ for Tpm3.1-actin, and 0.14 ± 0.06 s^−1^ for Tpm1.12-actin. Compared with the 2.1–3.6-fold reduction in the rate of the fast phase of ADP release, the rate of the slow phase was up to 23-fold reduced in the presence of Tpm. We measured values of 0.023 ± 0.001 s^−1^ for bare actin, 0.001 ± 0.0005 s^−1^ for Tpm1.8-actin, 0.007 ± 0.003 s^−1^ for Tpm3.1-actin, and 0.003 ± 0.0005 s^−1^ for Tpm1.12-actin (Fig. 3*C* and Table 2). Both the Tpm-induced acceleration in the rate of phosphate and the slower rate of ADP release contribute to an increase in the duty ratio, the fraction of time that NM-2B spends in strongly F-actin bound states during ATP turnover (52).

### Tpm-mediated changes in the motor activity and processivity of NM-2B

To analyze the Tpm-mediated changes of NM-2B motor activity directly, we performed *in vitro* motility assays. In the standard *in vitro* motility assay first described by Kron and Spudich (53), robust, unidirectional movement was observed. Compared with the situation with bare F-actin, the average unloaded velocity measured in this assay was 7, 28, and 35% slower in the presence of Tpm1.8, Tpm1.12, and Tpm3.1, respectively (Table 2).

**Figure 4.**
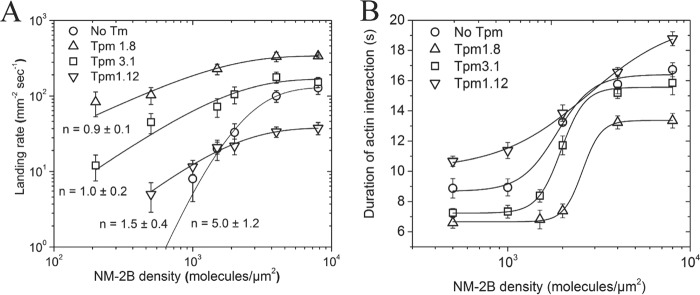
**Tpm-mediated changes in the frequency and duration of F-actin interactions with NM-2B.** *A*, the effect of the Tpm isoforms on the duty ratio of NM-2B was assessed by performing landing assays. Landing experiments were performed with a 60-fold stoichiometric excess of Tpm. Landing rates in the presence of ATP were recorded as a function of NM-2B surface density. Fitting of equation 1 to the experimental data gave values for *n*: 5.0 ± 1.2 for bare actin, 0.9 ± 0.1 with Tpm1.8-actin, 1.0 ± 0.2 with Tpm3.1, and 1.5 ± 0.4 with Tpm1.12-actin. *B*, the duration of actomyosin interactions during landing assays was assessed by determining the number of frames a given actin filament stays bound to myosin on the surface. Frames were recorded at intervals of 0.5 s. The data are expressed as the means ± S.E. Each point represents data obtained from three independent measurements.

To assess the extent to which Tpm-mediated changes of the duty ratio affect the motor properties of NM-2B, we performed landing assays. Actin filament recruitment was measured at varying NM-2B–HMM surface densities. The data were fitted using equation 1 (see “Experimental procedures”), where *n* approximates the minimal number of myosin molecules required to bind and propel actin filaments. Values of *n* close to 1 imply that only one motor molecule is required for processive movement along actin filaments. The measured values for n decrease from 5.0 ± 1.2 for bare-actin to 0.9 ± 0.1, 1.5 ± 0.4, and 1.0 ± 0.2 for Tpm1.8-, Tpm1.12-, and Tpm3.1-enveloped F-actin, respectively. Thus, the duty ratio of NM-2B increases from ∼20% with bare F-actin to 67% with Tpm1.12 and close to 100% with both Tpm1.8 and Tpm3.1. Compared with the situation with bare F-actin, the presence of each of the Tpm isoforms led to a large increase in the landing rate at low NM-2B surface densities. In the presence of Tpm1.8 or Tpm3.1, the number of observed landing events was also significantly increased at NM-2B surface densities greater than 1000 molecules/μm^2^. In contrast, Tpm1.12 caused a large drop in the landing rate at high NM-2B surface densities (Fig. 4*A*). Landing events were further analyzed to determine how the myosin density affects the duration of interactions between F-actin and NM-2B. Fig. 4*B* shows that Tpm3.1 and Tpm1.8 increase the cooperativity of NM-2B binding to F-actin but reduce the duration of the interactions. Tpm1.12-enveloped F-actin has the opposite effect. The cooperativity of NM-2B binding to Tpm1.12-enveloped F-actin is reduced, but once NM-2B has bound Tpm1.12-enveloped F-actin, it remains associated for significantly longer periods.

### Tpm decoration affects the run-length of NM-2B on β-actin filaments

**Figure 5.**
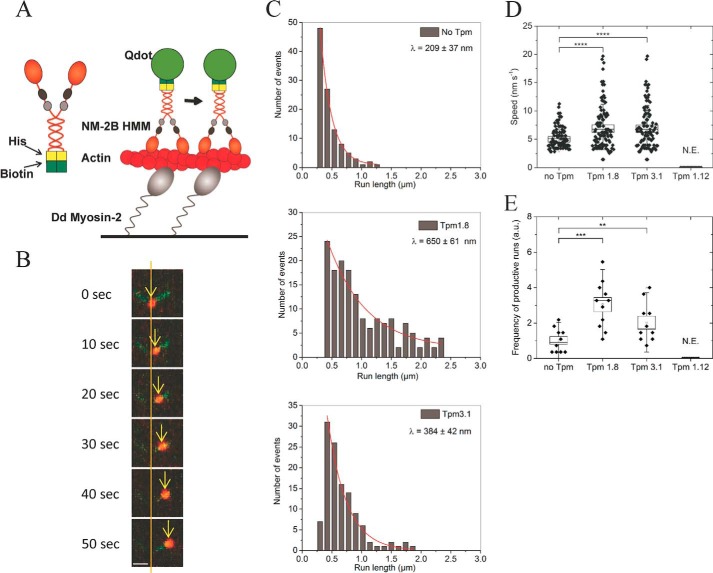
**Tpm-mediated changes in NM-2B motor activity.** *A*, schematic representations of the domain structure of the NM-2B–HMM construct and the experimental setup. *Dictyostelium discoideum* myosin-2 inactivated by *N*-ethylmaleimide treatment was utilized to anchor β-actin filaments to the glass surface. Rhodamine-phalloidin was used for stabilization and fluorescence labeling of the filaments. The movement of NM-2B–HMM was monitored. *B*, time course of a Qdot-labeled NM-2B–HMM (*red*) moving processively on a Tpm3.1–β-actin filament (*green*). The *scale bar* represents 2 μm. The *arrows* indicate the movement of the center of the Qdot. *C*, Tpm-mediated changes in the run length of NM-2B–HMM. Histograms showing the changes in run length for bare, Tpm1.8- and Tpm3.1-enveloped β-actin filaments. The characteristic run length, [lambda], was obtained by fitting *y* = *A* × *e*^−^*^x^*^/λ^ to the run length histogram. No runs were observed for Tpm1.12-enveloped filaments. *D*, scatter plots showing the velocity of NM-2B–HMM-Qdots on bare and Tpm-containing tracks of β-actin (*n* = 102, bare actin; *n* = 101, Tpm1.8-actin; *n* = 94, Tpm3.1-actin). The *box-and-whisker plots* represent standard error and standard deviation, respectively. The differences between bare actin and Tpm1.8-decorated actin, as well as bare actin and Tpm3.1-decorated actin are highly significant (****, *p* = 0.000043 and *p* = 0.000048, respectively). *E*, Tpm-induced changes in event frequency. The frequency of processive NM-2B–HMM runs is shown as normalized value, where the mean frequency of events on bare β-actin filaments corresponds to 1. No events were observed for Tpm1.12-enveloped actin. The *box-and-whisker plots* represent standard error and standard deviation, respectively. The observed differences are significant (*p* = 0.026 for no Tpm *versus* Tpm3.1 and *p* = 0.0004 for no Tpm *versus* Tpm1.8). *N.E.*, no events; *a.u.*, arbitrary units.

To directly evaluate the processivity of NM-2B on bare and Tpm containing β-actin filaments, we performed single-molecule motility assays using a quantum dot (Qdot)-labeled NM-2B–HMM-construct (Fig. 5*A*). The NM-2B–HMM construct displays short processive runs on bare actin filaments, with an average run distance of 209 ± 37 nm (*n* = 108). Tpm1.8- and Tpm3.1-enveloped F-actin supported an average run length of 650 ± 61 nm (*n* = 110) and 384 ± 42 nm (*n* = 112), respectively (Fig. 5, *B* and *C*). Assuming a step size of ∼5.5 nm, the longest processive runs recorded with Tpm1.8-enveloped F-actin correspond to nearly 500 consecutive steps. In the presence of Tpm3.1, we observed run lengths consistent with more than 300 consecutive steps. The velocity of the Qdot-labeled NM-2B–HMM construct showed significant increases by ∼30% in the presence of Tpm1.8 and Tpm3.1 (Fig. 5*D*). The frequency of productive runs increased 3-fold in the presence of Tpm1.8 and 2-fold for Tpm3.1. No processive runs were observed on Tpm1.12-enveloped filaments (Fig. 5*E*). Differences in motor activity observed between the standard and single molecule *in vitro* motility assays reflect changes in the number of interacting myosin heads, drag force, and mode of surface attachment.

## Discussion

Cell biological, structural, and biochemical studies lend support to the notion that Tpm isoforms contribute or even play an essential role in specifying the functional properties of cytoskeletal actin filament populations (9, 30, 41, 54). Our results show how different Tpm isoforms that coexist within the same cell type affect myosin motor activity. The fact that cytoskeletal actin filaments are mostly associated with tropomyosin cofilaments stresses the need for a conceptual change in the cytoskeletal actomyosin field. Namely, it requires a shift away from a generic view of the actin filament and toward a greater consideration for the properties of distinct cellular actin filament populations. Our results show the extent to which product release steps and the duty ratio of the myosin motor are affected by the association of F-actin with different Tpm isoforms. Dependent on the composition of the actin-Tpm cofilaments, the same cytoskeletal myosin motor is able to switch function from efficient transporter to tension holder, force sensor, or processive signal transducer.

NM-2B displays slow actin-activated ATP turnover and sliding velocity (51, 55). During basal ATP turnover, the release of the hydrolysis product P_i_ is the rate-limiting step of the NM-2B ATPase cycle. Similar to most myosins, P_i_ release is greatly accelerated by F-actin binding. However, unlike most myosins, binding to F-actin has been reported to slow the release of the second hydrolysis product ADP from the active site of NM-2B (51, 56). This type of negative coupling between actin binding and ADP release has also been observed with human myosin-7A, but is uncommon for other myosins (57). The force generating interaction of NM-2B with bare F-actin creates a situation where the release rates for both hydrolysis products become similar and contribute to limiting the rate of the overall cycle.

Our results show that all three Tpm isoforms bind NM-2B-decorated F-actin with affinities that are of physiological significance and affect the balance between P_i_ and ADP release from the active site of NM-2B in a specific manner. The associated changes in dominant rate-limiting steps have a direct effect on the duty ratio, because P_i_ release gates the transition from weak to strong F-actin binding states, and ADP release does the opposite. The duty ratio is increased ∼3-fold in the presence of Tpm1.12 and 5-fold for both Tpm1.8 and Tpm3.1. In regard to their effect on the time required per ATP turnover, the presence of Tpm1.12 extends the cycle time 3.7-fold, whereas it is shortened by 27 and 63% in the presence of Tpm 1.8 and Tpm3.1, respectively.

Tpm-induced changes in the second-order rate constant for NM-2B binding to F-actin and the first-order rate constant for dissociation from F-actin result in 2.9- and 2.2-fold greater affinity for F-actin in the presence of Tpm1.8 and Tpm1.12. The largest changes were observed in the presence of Tpm3.1, which resulted in a 9-fold greater affinity for F-actin. The combined effects of the observed changes in turnover rate, duty ratio, and actin affinity brought about by each of the Tpm isoforms can be probed using *in vitro* assays that monitor the functional competence of the myosin motor. Previously, Rock and co-workers (58) used a three-bead optical trapping assay to show that NM-2B dimers bind to filamentous α-actin and cause multiple displacements before detachment. In contrast, Sellers and co-workers (55) reported that only short bipolar NM-2B filaments move processively on filamentous α-actin. This apparent difference in processive behavior may be explained by the force-dependent ADP release kinetics described by both the Sellers and Rock groups (36, 69). It is also compatible with the results obtained in the landing assays, where NM-2B moving against zero loads on bare actin appears to require multiple molecules for continuous motion, whereas the increased Stokes drag associated with the attachment of an 18-nm Qdot to the C terminus of NM-2B–HMM appears to facilitate processive movement in our single myosin molecular experiments. A Qdot-mediated increase in Stokes drag with the associated decrease in diffusion rate along DNA has previously been reported for the motion of Qdot-labeled DNA *N*-glycosylases belonging to the helix–hairpin–helix and Fpg/Nei families (59). As expected from our transient kinetics results, NM-2B performs longer runs in the TIRF single-molecule motility assay on Tpm1.8– or Tpm3.1–β-actin cofilaments than on bare filaments. Interactions of NM-2B with Tpm1.12–β-actin cofilaments lasted typically three times longer but did not result in active translocation of the motor along the actin filament. Our kinetic data do not predict that at NM-2B surface densities above 1500 molecules/μm^2^, the number of landing events is lower in the presence of Tpm1.12 than with bare F-actin. In contrast to the two other Tpm isoforms, the association of Tpm1.12 with β-actin does not increase the cooperativity of NM-2B-binding to F-actin but rather decreases it. The major effect of Tpm1.12 appears to be that it gears the activity of the NM-2B motor toward tension-bearing rather than contractile functions. NM-2B is less likely to bind Tpm1.12–β-actin cofilaments in the crowded environment of actin arcs or stress fibers. However, once it binds, it remains strongly attached for longer periods. The opposite is true for Tpm 1.8 and Tpm3.1. Decoration of β-actin filaments with one of these Tpm isoforms increases the motile activity of NM-2B, its frequency of productive interactions, and ability to take multiple steps against an external force. The specific changes brought about by association with Tpm 1.8 or Tpm3.1 extend the functional range of NM-2B, within both the contexts of bipolar thick filaments and activated NM-2B monomers (33).

In summary, our work shows the extent and scope of the functional changes that are introduced by the decoration of cytoskeletal actomyosin complexes with different cytoskeletal tropomyosin isoforms. Future challenges include the elucidation of the composition of additional actin–Tpm–myosin complexes, their involvement in specific cellular tasks, and how their function and structural integrity are affected by post-translational modifications.

## Experimental procedures

### Neuroblastoma cell culture and immunofluorescence microscopy

SK-N-BE(2) cells were obtained from Dr. B. Förthmann (MHH, Hannover, Germany). The cells were maintained in Corning plastic ware in DMEM/F12 medium supplemented with 10% FCS, glucose (4.5 g/liter), sodium pyruvate (1 mm), and penicillin-streptomycin (100 units/ml). The cells were seeded on 12-mm glass coverslips and differentiated with 10 μm all-*trans*-retinoic acid for a period of 6 days. The cell were fixed with 1% PFA in incomplete DMEM, permeabilized for 5 min in 0.1% Triton X-100, and blocked for 1 h with 2% BSA in PBS. Samples were incubated with rabbit polyclonal NM-2B primary antibody (Covance, catalog no. PRB-445P) for 1 h at room temperature in blocking solution, in combination with the following sheep polyclonal Tpm specific antibodies: γ/9d (Merck Millipore, catalog no. AB5447), α/1b (Merck Millipore, catalog no. ABC499), and α/9d (Merck Millipore, catalog no. AB5441) (1). Secondary antibodies were applied for 30 min at room temperature in blocking solution: GAR-IgG-AlexaFluor488 (Jackson ImmunoResearch, catalog no. 111-545-144) and DAS-IgG-Cy3 (Merck Millipore, catalog no. AP184C). Images were acquired using a Leica TCS SP8 confocal laser microscope equipped with a 63×/1.4 NA objective (Research Core Unit for Laser Microscopy, MHH). Image analysis was performed with the Fiji release of ImageJ software version 1.49s (59).

### Cell lysis and immunoblot analysis

SK-N-BE(2) cells differentiated with 10 μm all-*trans*-retinoic acid for a period of 6 days were lysed in SDS sample buffer at a concentration of 2 × 10^4^ cell/μl. Lysates were separated on 12% acrylamide gels by SDS-PAGE. Samples were transferred onto a nitrocellulose membrane, blocked for 1 h at room temperature in 5% skim milk powder and labeled overnight with the following Tpm-specific sheep polyclonal antibodies: γ/9d (Merck Millipore, catalog no. AB5447), α/1b (Merck Millipore, catalog no. ABC499), and α/9d (Merck Millipore, catalog no. AB5441) (1). A donkey anti-sheep IgG-HRP secondary antibody (Santa Cruz Biotechnology, catalog no. sc-2473) was applied for 1 h at room temperature before HRP activity was developed using the SuperSignal^TM^ West Femto maximum sensitivity substrate (Thermo Fisher, catalog no. 34095). Membranes were imaged with a Bio-Rad ChemiDoc^TM^ MP system using Image La^TM^ software (Bio-Rad).

### Cloning, expression, and protein purification

To generate the expression construct for the heavy chain of the double-headed NM-2B–HMM construct with C-terminal octahistidine and Avi tags, the DNA sequence that encodes amino acids 1–1344 was PCR-amplified using human cDNA as a template. The primers used were 5′-CGCGCGGCGCGCATGGCGCAGAGAACTGGACTCGA-3′ and 5′-CAGCTGGTCGACGTGGTGATGATGATGATGATGATGCTCCTCTTCCAGCTGCCGGAT-3′ containing restriction sites for BssHI and SalI, respectively. The pFast Bac^TM^ vector containing the NM-2B heavy chain sequence and the pFastBac^TM^ Dual vector containing the sequences for MYL6 and MYL12b were cotransformed for protein production in SF9 cells. Vectors and cells were obtained from Thermo Fisher Scientific. The generation of the expression vector encoding the motor domain of human NM-2B0 (residues 1–782), two *Dictyostelium discoideum* α-actinin repeats, and a C-terminal octahistidine tag was described elsewhere (47). Sf9 cells were transfected with the recombinant bacmids using the FuGENE HD transfection reagent (Promega). To purify the free MYL6 and MYL12b light chains, the genes were cloned into the pGEX expression vector (GE Healthcare Life Sciences) for protein production in *E. coli*.

Expression constructs for rat Tpm1.8cy (b.–.b.d) (NCBI reference ID NP_001029245.1), rat Tpm1.12br (b.–.b.c) (NCBI reference ID NP_001288665.1), and human Tpm3.1cy (b.–.a.d) (NCBI reference ID NP_705935.1) were cloned into pET23a^+^ for expression in *E. coli* strain Rosetta^TM^ (Merck). We refer to the different tropomyosin isoforms in the text mostly by their short name in accordance with a recently introduced systematic nomenclature (60). Full formal protein names are used when we refer to differences in exon usage between the isoforms. Alternate names and changes in exon usage are listed in Table 1. Moreover, we refer to the unassembled double-stranded coiled-coil protein as dimer. Cytoskeletal Tpm isoforms form predominantly homopolymers of Tpm homodimers. The extensions “br” and “cy” in the formal protein name indicate prominent but not exclusive associations with brain tissue and cytoskeletal structures. Tpm1 or Tpm3 specifies the protein is encoded by TPM1 or TPM3. The four-letter code extension of the formal protein name indicates splice form usage for the variable exons 1, 2, 6, and 9. All three isoforms lack the region encoded by exon 2 (60). The short names Tpm1.8, Tpm1.12, and Tpm3.1 are used throughout the text, unless we refer to the splicing of the four exons that vary in vertebrates.

Purification of chicken skeletal muscle α-actin (61) and recombinant human β-actin was performed as described previously (62). Pellets of *Sf9* cells producing NM-2B0 motor domain or HMM constructs were suspended in lysis buffer (50 mm HEPES, pH 7.5, 200 mm NaCl, 10 mm β-mercaptethanol, 4 mm MgCl_2_, 4 mm ATP, 1 mm EGTA, 0.5% Triton X-100, and cOmplete^TM^ protease inhibitor mixture (Merck). The cell suspension was sonicated four times for 1 min at 40% power and 50% duty cycle (Bandelin Sonoplus, Berlin, Germany). The lysate was centrifuged at 100,000 × *g* for 1 h at 4 °C. The supernatant was loaded onto a Ni^2+^–nitrilotriacetic acid affinity column (Qiagen) equilibrated with lysis buffer. The column material was washed with 10 column volumes lysis buffer and 10 column volumes wash buffer (25 mm HEPES, pH 7.3, 200 mm NaCl, 0.5 mm EGTA, 3 mm MgCl_2_, 65 mm imidazole). The protein was eluted with 6 column volumes elution buffer (25 mm HEPES, pH 7.3, 200 mm NaCl, 1 mm EGTA, 1 mm EDTA, 1 mm DTT, and 200 mm imidazole). In addition, the free forms of human recombinant ELC (essential myosin light chain) (*MYL6*) and RLC (regulatory myosin light chain) (*MYL12b*) constructs were produced in *E. coli*. The purified light chains were added at 1 μm concentration to the elution buffer used for the purification of the HMM construct. Individual fractions were analyzed by SDS-PAGE, concentrated, and dialyzed overnight against dialysis buffer (25 mm HEPES, pH 7.3, 200 mm NaCl, 1 mm EGTA, 1 mm EDTA, 1 mm DTT, and 3% trehalose). Dialyzed protein was applied to a HiLoad Superdex 200 prep grade 16/60 gel filtration column (GE Healthcare Life Sciences) equilibrated with dialysis buffer. The resulting protein fractions were concentrated to a final concentration of ∼6 mg ml^−1^. The purified protein was supplemented with 7% (w/v) trehalose, flash frozen in liquid nitrogen, and stored at −80 °C.

Tpm production was induced by the addition of 1 mm isopropyl β-d-thiogalactopyranoside, followed by incubation with vigorous shaking for 3–4 h at 37 °C. In addition to human Tpm3.1, we produced rat isoforms Tpm1.8 and Tpm1.12. Changes between rat and human Tpm1.8 and Tpm1.12 occur at S30T, Q37H, and K184R. In the case of Tpm1.12, a fourth change corresponds to H226Q. All changes occur in the c position of the repeating heptad repeat pattern (63). Residues in the c position are located at the outside of the coiled-coil and contribute to stability through α-helical propensity (64). The program Paircoil2 (43) predicts the parallel coiled-coil fold of Tpm1.8 and Tpm1.12 to be unchanged by these species specific substitutions. The recombinant, tag-free Tpm constructs were purified by ion exchange chromatography as described by Coulton *et al.* (65) with minor modifications. Purified Tpm was stored in 5 mm potassium phosphate, pH 7.0, 100 mm NaCl, and 5 mm MgCl_2_. Protein concentration was determined using the Bradford assay and protein absorbance readings at 276 nm. Measurements were performed with a UV-2600 spectrophotometer (Shimadzu, Duisburg, Germany). Recombinant myosin light chain kinase was obtained from Sigma–Aldrich.

### Binding assays

The affinity of Tpm1.8, Tpm1.12, and Tpm3.1 for filamentous β-actin was measured and analyzed using a cosedimentation assay as described by Coulton *et al.* (65). 10 μm β-actin was preincubated with varying Tpm concentrations (0–10 μm) for 30 min at room temperature. Assay buffer contained 20 mm MOPS, pH 7.0, 5 mm MgCl_2_, and 100 mm KCl. NM-2B-induced Tpm binding to β-actin was monitored following the cosedimentation protocol described by Moraczewska *et al.* (25). The 6:1 acto–Tpm complex was mixed with increasing concentrations of NM-2B (0–16 μm), incubated for 30 min in assay buffer (20 mm MOPS, pH 7.0, 5 mm MgCl_2_, and 50 mm KCl), and pelleted for 20 min at 100,000 × *g*. To increase the sensitivity and linearity of the cosedimentation assays, supernatant and pellet fractions were treated with Instant-Bands sample loading buffer (EZBiolab Inc., Carmel, IN) and resolved by SDS-PAGE. Quantification of band intensities was performed using densitometry on a Bio-Rad ChemiDoc^TM^ MP system (Bio-Rad). The images were analyzed with the Fiji release of ImageJ software version 1.49s (59).

### Kinetic measurements

NM-2B–HMM was phosphorylated immediately prior to use at 30 °C for 30 min. NM-2B–HMM was incubated with myosin light chain kinase at a stoichiometric ratio of 20:1 in a reaction mixture containing 2 μm RLC, 20 mm MOPS, pH 7.0, 50 mm KCl, 2 mm MgCl_2_, 1 mm CaCl_2_, 0.15 mm EGTA, 0.2 μm calmodulin, 2 mm DTT, and 1 mm ATP. The single-headed NM-2B0 construct does not require activation and was used without prior phosphorylation.

Steady-state ATPase rates were measured using an enzyme-based assay, linking ATP hydrolysis to the oxidation of NADH. The change of absorbance was followed over 30 min at 25 °C in assay buffer containing 25 mm HEPES, pH 7.4, 5 mm MgCl_2_, and 50 mm KCl. Increasing actin concentrations (0–50 μm) were preincubated with a 3-fold molar (18-fold stoichiometric) excess of Tpm for 30 min at 23 °C. Values for the maximum actin-activated ATPase activity (*k*_cat_) and the actin concentration required for half-maximal activation (*K*_app_), and the apparent second-order rate constant for F-actin binding (*k*_cat_/*K*_app_) were determined as described previously (57).

Transient kinetic experiments were performed at 20 °C in either a HiTech Scientific SF- SF-61 SX or SF-61 DX stopped-flow system. Both systems are equipped with a 75 W mercury–xenon arc lamp. The assay buffer used contained 20 mM MOPS, pH 7.0, 100 mM KCl, and 5 mM MgCl_2_. In the case of Tpm-enveloped actin filaments, the complex of actin and Tpm was preincubated for at least 30 min at 23 °C.

The binding of NM-2B to actin was measured by following the increase in light scattering at 405 nm occurring upon mixing 4 μm NM-2B with 0.5–4 μm actin or actin-Tpm complex. The P_i_ release assay was performed as described previously (66, 67). First, 2 μm NM-2B was mixed with 1 μm ATP. This was followed by incubation for 15 s in a delay line to allow ATP binding and hydrolysis to occur. In the second mixing step, the release of phosphate was triggered by the addition of 40 μm actin or actin-Tpm, where Tpm constructs were added in a 3-fold molar excess over actin. Binding of P_i_ to *N*-[2-(1-maleimidyl)ethyl]-7-(diethylamino)coumarin-3-carboxamide–labeled phosphate-binding protein was monitored.

The rate of ADP release from the HMM construct was determined as described by Sellers and co-workers (51). The decrease in dmADP fluorescence upon mixing acto-NM-2B–HMM complexes with a large excess of unlabeled ADP in the presence or absence of Tpm was measured. Premix concentrations in the stopped-flow apparatus correspond to 0.4 μm myosin heads, 10 μm dmADP, 2 μm actin, 2 mm ADP, and either 0 or 20 μm Tpm construct.

Kinetic Studio software (TgK Scientific, Bradford on Avon, UK) was used for initial data inspection and analysis. Detailed data analysis was performed with OriginPro (Northampton, MA) 9.0G graphing and data analysis software. Goodness-of-fit criteria were evaluated using the coefficient of determination *R*^2^ and χ^2^ tests as implemented in OriginPro 9.0G. Each data point corresponds to the average of 3–6 single measurements and was verified at least once with protein from different preparations. The *error bars* indicate the standard deviation.

### Assays for actin sliding movement

Unloaded actin sliding motility was measured at 30 °C as described previously (53). HMM was directly bound to the nitrocellulose-coated surface. Actin filament velocity was determined with the help of the program DiaTrack 3.01 (Semasopht, Switzerland). Data analysis was performed with Origin 9.0G (OriginLab). Goodness-of-fit criteria were evaluated using the coefficient of determination *R*^2^ and χ^2^ tests as implemented in Origin 9.0G.

Landing assays were performed as described previously (68) with the following modifications: NM-2B–HMM molecules were directly immobilized on nitrocellulose-coated coverslips to obtain surface densities between 200 and 8,000 myosin molecules/μm^2^ (69). The assay was started by the addition of TRITC-phalloidin-labeled actin (20 nm, with and without decoration with Tpm) to the motility buffer containing 4 mm Mg^2+^-ATP. Landing events were recorded in TIRF mode using a customized inverted microscope (Olympus IX81) equipped with a 532 nm diode laser (Novalux, Sunnyvale, CA) and fitted with a 60×/1.49 NA oil immersion lens (ApoN, Olympus). The landing rate was measured by counting the number of actin filaments that landed and moved ≥0.3 μm in an observation area of ∼7,100 μm^2^. The density of myosin motors on the assay surface (molecules/μm^2^) is plotted against the observed landing rate (mm^−2^ s^−1^). Landing rates were best fit to Equation 1 according to the model by Hancock and Howard (68).

(Eq. 1) $$R_{\text{n}}\left(\rho \right)=Z{\left(1-e^{-\frac{\rho }{\rho_{0}}}\right)}^{n}$$

Here, *Z* is a parameter that incorporates collision of actin with the surface, and ρ_0_ corresponds to the surface area over which motors interact with actin. The duty ratio is given by the slope *n*, which can be obtained from the graph. At *n* = 1, only one motor is required to propel an actin filament forward.

### Determination of processive run length

To investigate the run lengths of NM-2B motors, biotinylated NM-2B–HMM was mixed with a 10–20-fold molar excess of 655-nm streptavidin-coated Qdots (Invitrogen), which ensures that the majority of motile Qdots are bound to a single NM-2B–HMM. Biotinylation of the C-terminal AviTag of NM-2B–HMM was performed, along with RLC phosphorylation. Following the addition of 50 μm biotin, 0.6 μm BirA, and 0.005 μm myosin light chain kinase to 1 μm NM-2B–HMM (final concentrations) in buffer containing 25 mm imidazole, 1 mm CaCl_2_, 0.15 mm EGTA, 0.2 μm calmodulin, 1 mm DTT, and 1 mm ATP, the reaction mixture was incubated at 30 °C for 30 min. Flow cells made from glass coverslips were prepared by introducing the following solutions into the flow cell: 0.05 mg/ml anti-His antibody (5 min incubation) and 0.2 mg/ml *N*-ethylmaleimide-modified *D. discoideum* myosin-2 motor domain construct with artificial lever arm and C-terminal His tag (70). Incubation for 2 min was followed by a wash with buffer containing 1 mg/ml BSA and 1 mm DTT, incubation with rhodamine-phalloidin-labeled β-actin filaments with or without Tpm (2–5 min), and a wash with motility assay buffer. Finally, we added 0.1 μm NM-2B with 1 or 2 μm Qdots in motility buffer containing 4 mm Mg-ATP and an oxygen-scavenging system (36). For experiments with actin-Tpm, a very large excess of Tpm (10 μm) was included in the motility buffer to prevent dissociation from F-actin. TIRF fluorescence microscopy was performed at room temperature (22 °C) using a Nikon Eclipse Ti spinning disc microscope equipped with a 100× TIRF Apo objective lens (1.49 NA). Qdots and TRITC-phalloidin–labeled actin were excited with the 488- and 561-nm laser lines, respectively. Images were acquired simultaneously using a two-camera adaptor and Andor iXon Ultra EMCCD cameras. The pixel resolution was 76.0 nm, and data were collected at 60 frames/min. Qdot movement along actin filaments was tracked manually using ImageJ. For each event, we required Qdot-labeled NM-2B–HMM to move continuously for at least five frames to qualify as a run. Runs that artificially terminated by running off the end of an actin filament and runs shorter than 2 pixels were not included in the run-length analysis.

### Statistical analyses

The data are expressed as the means ± S.E. Student's paired *t* test (2-tailed) was applied to determine the significance of the differences between bare and Tpm1.8-, Tpm1.12-, or Tpm3.1-enveloped actin filaments. Statistical significance is assigned follows; *ns*, *p* > 0.05; *, *p* ≤ 0.05; **, *p* ≤ 0.01; ***, *p* ≤ 0.001; ****, *p* ≤ 0.0001.

## Author contributions

S. P.-C. and T. R. purified proteins, performed experiments, and analyzed data; M. H. T. performed experiments and analyzed data; N. H. produced the NM-2B–HMM plasmid construct and made contributions to the experimental design; S. L. L. performed cellular and immunofluorescence experiments and analysis and contributed Fig. 1; D. J. M. conceived and coordinated the study, advised S. P.-C., M. H. T., T. R., N. H., and S. L. L., wrote the manuscript, and was responsible for funding acquisition and project administration. All authors contributed by drafting the article and revising it critically.

## Footnotes

4 The abbreviations used are:

Tpm: tropomyosin
NM: nonmuscle myosin
dmADP: 2′-deoxy-3′-mant-ADP
Qdot: quantum dot
TRITC: tetramethylrhodamine isothiocyanate
HMM: heavy meromyosin
MHH: Medizinische Hochschule Hannover
TIRF: total internal reflection fluorescence.
